# Analysis of sociodemographic, clinical, and lifestyle factors associated with cognitive aging

**DOI:** 10.1192/j.eurpsy.2023.415

**Published:** 2023-07-19

**Authors:** A. Suwalska, W. Pałys, D. Łojko, J. Suwalska, S. Tobis, M. Kasierska, K. Wieczorowska-Tobis

**Affiliations:** ^1^Department of Mental Health, Poznan University of Medical Sciences, Poznan; ^2^Faculty of Health Sciences, Academy of Kalisz, Kalisz; ^3^Department of Adult Psychiatry; ^4^Department of Treatment of Obesity, Metabolic Disorders and Clinical Dietetics; ^5^Department of Occupational Therapy; ^6^Department of Palliative Medicine, Poznan University of Medical Sciences, Poznan, Poland

## Abstract

**Introduction:**

Cognitive aging is defined as the cognitive decline during the aging process. Most cognitive skills deteriorate in old age; however, there are individual differences in the speed of the decline and severity of neuropsychological deficits.

**Objectives:**

The aim of the study was to delineate the associations of sociodemographic, clinical, and lifestyle factors with cognitive aging.

**Methods:**

302 participants aged 60 years and above (mean age 69,6±7,2; range 60-92 years) were included in the study. Women were 69.9% of the group (N = 211). Subjects completed the questionnaire (sociodemographic and anthropometric data, chronic diseases), and depression intensity was assessed by Beck Depression Inventory (BDI). Cognitive functions were evaluated using MiniMental State Examination, Trail Making Test, Stroop test, and selected tests from CANTAB battery (Pattern Recognition Memory, Spatial Recognition Memory, Spatial Span, Spatial Working Memory).

**Results:**

Age influenced most of the studied cognitive functions. Higher education level and more frequent cognitive activities (e.g. reading and crosswords) had a protective effect on the performance of tests assessing working memory and executive functions. Working memory and attention assessed in the Stroop test in part B were most sensitive to the negative impact of age, lower education level, and lower frequency of cognitive activity. Higher body mass index (BMI≥28) and diabetes were associated with worse spatial working memory.

**Conclusions:**

The results suggesting the association between lifestyle factors – cognitive activity and cognitive functions can contribute to the development of interventions aimed at the preservation of cognitive functions in older age.

**Disclosure of Interest:**

None Declared

